# The impact of income-support interventions on life course risk factors and health outcomes during childhood: a systematic review in high income countries

**DOI:** 10.1186/s12889-023-15595-x

**Published:** 2023-04-22

**Authors:** Delia Boccia, Silvia Maritano, Costanza Pizzi, Matteo G. Richiardi, Sandrine Lioret, Lorenzo Richiardi

**Affiliations:** 1 Faculty of Population and Health Policy, London School of Hygiene and Tropical Medicine, 15-17 Tavistock Pl, London, WC1H 9SH UK; 2 Department of Medical Sciences, University of Turin and CPO-Piemonte, Turin, Italy; 3 University School for Advanced Studies IUSS Pavia, Pavia, Italy; 4 Centre for Microsimulation and Policy Analysis, Institute for Social and Economic Research, University of Essex, Colchester, UK; 5 Université Paris Cité, INSERM, INRAE, Paris, CRESS France

**Keywords:** Child health, Inequalities, Social support, Income, Social protection, Life-course

## Abstract

**Background:**

In high income countries one in five children still lives in poverty, which is known to adversely shape the life course health trajectory of these children. However, much less is understood on whether social and fiscal policies have the capacity to reverse this damage, which intervention is likely to be most effective and when these interventions should be delivered to maximise their impact. This systematic review attempts to address these questions by looking at the impact of income-support interventions, delivered during the first 1,000 days of life, on cardiovascular, metabolic, respiratory and mental health outcomes.

**Methods:**

The review was restricted to experimental or quasi experimental studies conducted in high income countries. Studies were retrieved from multidisciplinary databases as well as health, economic, social sciences-specific literature browsers. All papers retrieved through the search strategy were double screened at title, abstract and full text stage. Relevant data of the selected studies were extracted and collected in tables, then summarised via narrative synthesis approach. Robustness of findings was assessed by tabulating impact by health outcome, type of intervention and study design.

**Results:**

Overall, 16 relevant papers were identified, including 15 quasi-experimental studies and one randomized control trial (RCT). Income-support interventions included were unconditional/conditional cash transfers, income tax credit and minimum wage salary policies. Most studies were conducted in United States and Canada. Overall, the evidence suggested limited effect on mental health indicators but a positive, albeit small, effect of most policies on birth weight outcomes. Despite this, according to few studies that tried to extrapolate the results into public health terms, the potential number of negative outcomes averted might be consistent.

**Conclusions:**

Income-support interventions can positively affect some of the health outcomes of interest in this review, including birth weight and mental health. Given the large number of people targeted by these programs, one could infer that – despite small – the observed effect may be still relevant at population level. Nonetheless, the limited generalisability of the evidence gathered hampers firm conclusions. For the future, the breadth and scope of this literature need to be broadened to fully exploit the potential of these interventions and understand how their public health impact can be maximised.

**Supplementary Information:**

The online version contains supplementary material available at 10.1186/s12889-023-15595-x.

## Background

### Introduction

Despite the overall global improvement of most development indices, one in five children in high income countries still lives in poverty, with striking variation across countries in terms of prevalence [1]. In one recent analysis from UNICEF (United Nations International Children's Emergency Fund**)** involving 41 high income countries, Denmark showed the best record on relative poverty. However even in such an affluent country, 9.2 percent of children are considered poor (defined as living in a household with income below 60% of the median household after housing costs). Israel and Romania showed the worst records on relative poverty, with more than one child in three falling below the poverty line. Bulgaria, Mexico, Spain, Turkey and the United States also have child poverty rates substantially greater than the rich-world average [1]. Recently, some high income countries are witnessing a rise in childhood poverty: in the United Kingdom, for example, child poverty rose by two percentage points between 2014 and 2017. According to the most recent sources of data, the only year that child poverty levels were reduced by more than 1% since 2010 was in 2021, when a temporarily 20 GBP weekly increase to Universal credit was introduced [2].

These forecasts are likely to having been exacerbated by the COVID-19 pandemic [3]. A mounting body of evidence suggests unequivocally that exposure to adverse socioeconomic circumstances during foetal life and early childhood affects clinical, behavioural and cognitive outcomes and—most importantly—can shape later life health trajectories [4]. These socioeconomic inequalities are preventable and unfair, particularly in the case of children who have little control over their health and the factors that influence it [5].

Overall, strategies to prevent, reduce and mitigate child poverty and its consequences generally involve three key components—support of early childhood education and care, income redistribution through cash and or in-kind benefits and tax systems, and policies to increase the employment chances and wages of families living in poverty [6, 7]. These measures are considered to play a crucial role in reducing child health inequalities mainly by increasing children’s human capital, reducing their vulnerability to the financial and physical consequences of ill-health and overall by interrupting the intergenerational transmission of poverty.

While there is evidence that all three components are likely to be effective at reducing child poverty globally, [6] at least in high income countries, few experimental and quasi-experimental studies have sought to determine whether the poverty effect of these macro-level interventions translate into a positive child-health effect [8]. There is also limited understanding of what type of interventions and when during childhood they may exert the greatest impact and who is most likely to benefit from them.

Important knowledge gaps remain also in terms of: a) *how* socioeconomic disadvantage experienced during early childhood *biologically* affects individuals’ life course health trajectories; and b) the extent to which the biological damages are exerted by socioeconomic disadvantage and c) *how* these biological damages can be effectively prevented and/or repaired through interventions able to address income inequalities during the first 1,000 days of life (from pregnancy to age 2) [9].

This review aims to contribute to these knowledge gaps by providing an evidence synthesis of the child health impact of macro-level socioeconomic interventions, and in particular of income support policies, delivered in the 1,000 days of life. This effort is part of the LifeCycle project, funded by EU Horizon 2020 (2017–2022)., The LifeCycle scope is to leverage knowledge from a network of EU child cohorts in order to: 1) identify markers of early-life stressors affecting health throughout the life course, including socioeconomic, lifestyle, migration and urban environment ones, and 2) translate the findings into policy recommendations for targeted prevention strategies.

### The conceptual framework

**Fig. 1:**
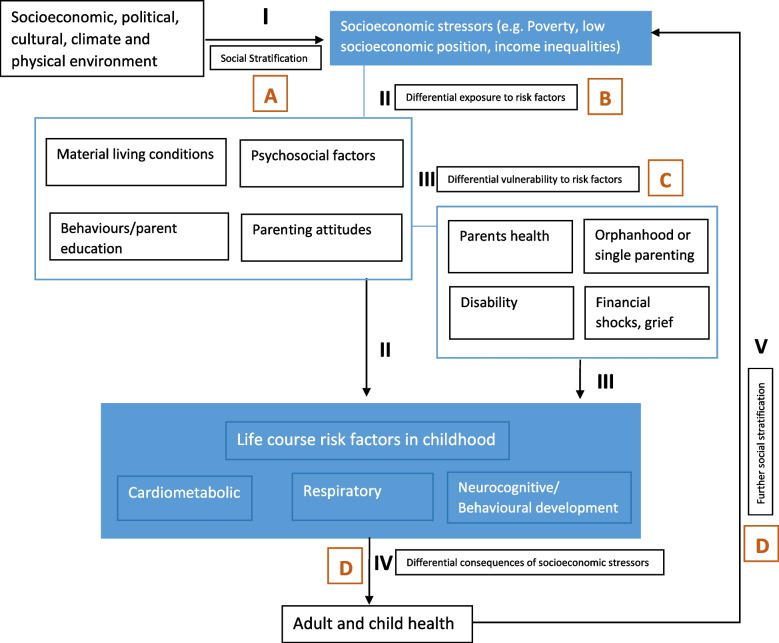
LifeCycle conceptual framework: impact of socio-economic stressors on life course risk factors

Early life socioeconomic stressors can affect life course cardiometabolic, respiratory and mental health outcomes through epigenetic mechanisms or by influencing both fetal and childhood development and adaptation, and the differential burden of risk factors and health outcomes during early life. In order to identify entry points for interventions, this framework needs to be further unpacked to elucidate the pathways through which socioeconomic disadvantage arises, operates and is perpetuated **(**Fig. 1**)**.

Following from Diderichsen and colleagues conceptual model, [5] we can assume that the primer drivers of socioeconomic stressors in childhood are positioned at distal level and refer to those structures and constructs that influence the socioeconomic position of individuals in a society (Fig. 1, Pathway I). Socioeconomic differences can influence the *differential exposure* to important material, psychosocial and behavioral risk factors (Fig. 1, Pathway II) or can affect the *differential susceptibility* of children to these risks (Fig. 1, Pathway III, e.g. the impact of any given risk factor may be more pronounced in less advantaged groups due to their greater likelihood of being exposed to other important and interacting risk factors). Finally, socioeconomic stressors may influence the *differential vulnerability* of children to the clinical and financial consequences of health conditions during childhood (Fig. 1, Pathway IV), which ultimately can further exacerbate the disadvantage in early life and adulthood (Fig. 1, Pathway V).

Depending on the pathway, we can identify different entry points for interventions as outlined in Table 1. For the purpose of this review, and consistent with the objectives of LifeCycle, we decided to concentrate on distal-level interventions that directly affect social stratification, and are aimed at reducing inequalities through educational, labor market, welfare and poverty alleviation strategies (Pathway I, Intervention A). Within this broad group of interventions, we focussed on income support interventions, defined as all measures taken by authorities and aimed at providing an adequate income to their citizens via different benefit schemes, which are implemented within different policies with different aims and objectives [10]. Their implementation may embrace different criteria of selectivity and generosity across each intervention and setting. Overall, two models were hypothesized to explain how income support programs might be able to improve child and adolescent outcomes. The first is the family investment model, according to which families have more money to spend on inputs [11, 12] or more time to spend with children [13]. The other hypothesized mechanism is the family stress model, according to which maternal depression and stress are lower because household resources are higher [14].

**Table 1:** Examples of entry points for interventions that address socioeconomic stressors in the early life

	**Pathway**	**Interventions**	
*I*	*Creation of social inequalities and disadvantage*	**A**	Policies that influence the process of social stratification through educational system, labour market, taxation and legislation, welfare and poverty-alleviation strategies
*II*	*Differential exposure to risk factors*	**B**	As above, but also policies that include classic public health interventions that improve housing, working conditions, and access to education and health services
*III*	*Increased vulnerability to risk factors*	**C**	Policies that include both social and public health intervention in a multisectorial/coordinated fashion to address the amplified health impact among children experiencing multiple risk factors at the same time

### Why this review is important

Recently, at least three systematic reviews have attempted to explore the child health impact of poverty alleviation strategies [15–17].

They differ from our own work in terms of scope and key inclusion criteria. The systematic review and meta-analysis from Courtin et al. [15] included a wide range of social policies/poverty alleviation strategies including housing, education and health insurance-related interventions, examined only RCTs, and looked at the impact on health in the general population rather than just on child’s health only.

Another recent review, from Cooper et al., [16] examined the effect of household income itself rather than socioeconomic interventions able to modify income and included as exposure of interest also lotteries and income shocks. They broadened their inclusion criteria to include findings from observational studies and extended their focus beyond strict health outcomes, including school achievement.

Finally, Simpson et al. [17] focused on the impact of social security benefits—more precisely the effect of changes in the eligibility and the amount and type of benefits provided -on a number of mental health indicators both in adults and children. Only observational studies were included in this review.

Our review adds to this body of knowledge by setting more stringent inclusion criteria and its boundaries of investigation. Specifically, this review aimed to generate evidence on the impact of interventions able to modify the effect of early-life socioeconomic stressors during the first 1,000 days of life. Moreover, in order to more specifically link and interpret our findings according to potential underlying mechanisms, we focused on those interventions that affected income inequalities. While the results of previous studies suggested some effects of socioeconomic policies on overall physical and mental health outcomes, we aimed to assess whether these interventions can affect children specific cardiovascular, metabolic, respiratory and mental health outcomes, relying only on results coming from either experimental or quasi experimental studies.

## Methods

All methods used in this review were carried out in accordance with relevant guidelines and largely followed the recommendations of Waddington et al. on the review of international development interventions [18]. With the exception of the search strategy definition and roll out, all steps were undertaken in parallel from at least two authors of this report.

### Search strategy and databases

Electronic searches have covered key bibliographic databases including:

- Multidisciplinary ones, such as SCOPUS, Web of Science and Google Scholar;
- Specific to social sciences, both general and discipline-specific, such as Social Science Research Network (SSRN), and Econlit for economics, PsycInfo for behavioural studies;
- Specific to biomedical research, including Pubmed/Medline, EMBASE;
- The Cochrane Library CENTRAL for both trials and reviews registry.

Consistent with existing recommendations, [18] we adopted a ‘snowballing’ approach: starting from important primary studies and already existing reviews we further increased the body of references both by bibliographic back-referencing and citation tracking (i.e. reviewing references in which the included study has been cited).

In terms of search strategy, we focused on two groups of key terms to begin with:

GROUP 1**-** Social welfare OR Social protection OR Cash/food/in-kind transfers OR child grants OR child benefits OR child allowances OR Income support OR Tax benefits OR Child tax credit;

GROUP 2 – child health.

Each term in GROUP 1 was cross-tabulated with all terms in GROUP 2. Given the broad scope of the review, we adopted an iterative process and refined the search strategy as we progressed. Key papers were also searched for in databases to identify subject headings or descriptors applied to them, which were then used to further refine the search strategy. The approaches above returned a final search strategy which is explained in Additional file 1. For all searches, high-income countries and RCT, experimental and quasi-experimental studies, filters were used. The electronic search was performed between October 2020 and February 2021 and further updated in August 2022.

### Eligibility criteria

Overall, only studies from high-income countries, as defined by the World Bank, which provided impact evidence of income-support interventions on the outcomes of interest were included in the review.

We included all macro-level interventions aimed at increasing income, i.e. income support intervention, among which:

- Social protection strategies (based on social assistance and safety nets, such as: conditional or unconditional cash transfers; price subsidies for electricity, public transport or food such as food stamps, vouchers, and coupons);
- Taxation policies and benefits (i.e. fee waivers and exemptions for schooling, tax credits, and utilities);
- Minimum wage salary policies.

We excluded interventions addressing differential exposure to risk factors (i.e. housing) and differential vulnerability to risk factors in disadvantaged groups (i.e. support for disabled people in the household). We also excluded school feeding programs as they were considered as a separate type of intervention, also typically delivered after the age window of interest. Finally, we excluded interventions that directly affected health outcomes (e.g. Medicaid or medical insurance-related interventions) because they could affect directly child health, beyond our conceptual framework pathways.

We considered interventions delivered during children’s first 1000 days of life only: this is a key period for determining lifetime health trajectories, since influences in early-life can cause long-term functional and structural changes [19].

Outcomes of interest included childhood life-course risk factors and health outcomes concerning:

- Cardiovascular health (e.g. specific diseases or parameters as blood pressure measurements);
- Metabolic conditions (e.g. birth weight, obesity, diabetes mellitus);
- Respiratory diseases: (e.g. Wheezing, Asthma, Chronic Obstructive Pulmonary Disease);
- Mental health: specific diagnoses (e.g. Attention Deficit Hyperactivity Disorder, Acute Stress Disorder, Internalizing/Externalizing behaviour problems) or self-assessment/reporting of mental health status.

Studies including impact on generic, self-reported measures of the overall health status were not included.

Finally, we applied restrictions on study design including only studies that reported impact evidence from Randomised Controlled Trials (RCTs) and Quasi-Experimental design studies. No time or language restriction was applied to papers.

### Studies selection

**Fig. 2:**
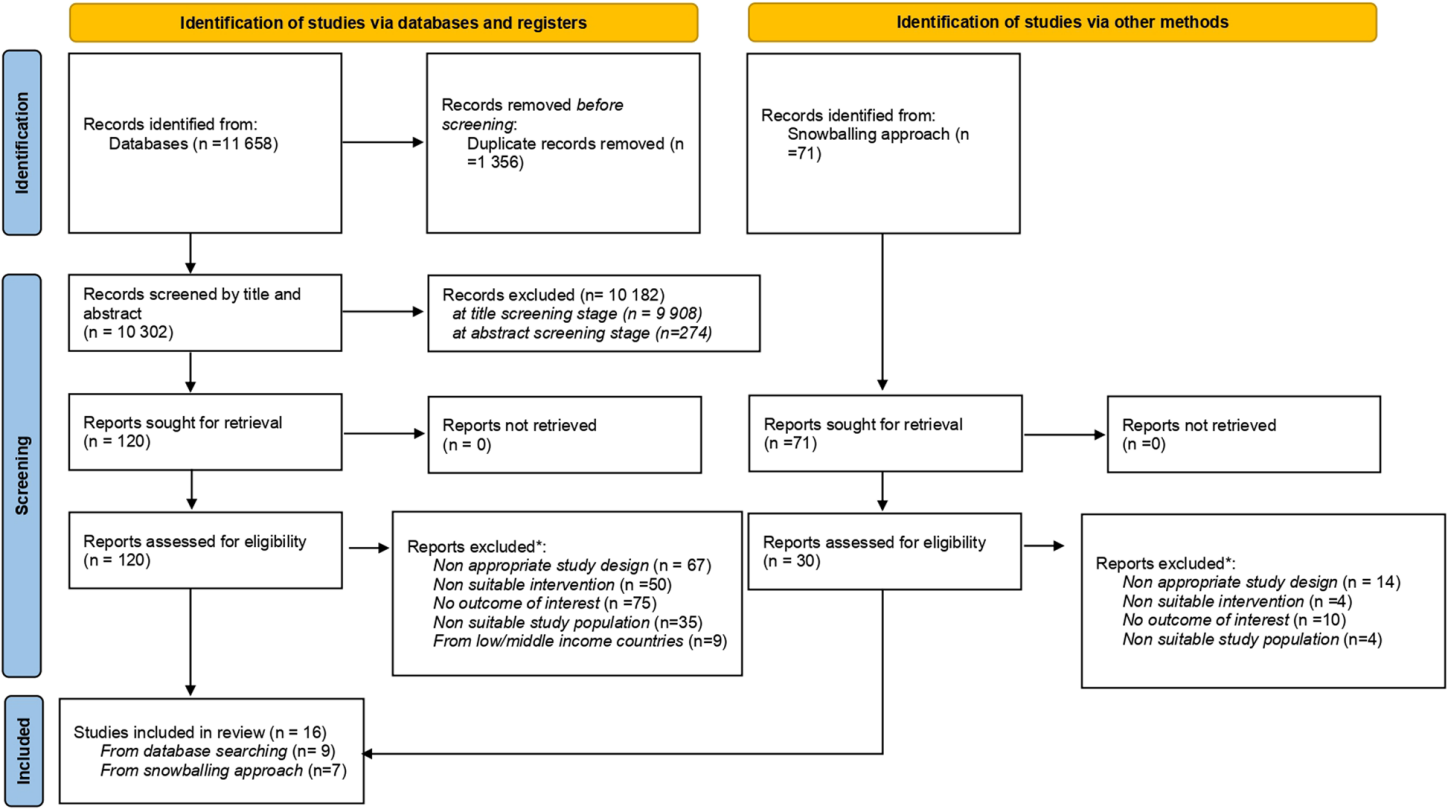
PRISMA flow diagram of studies include/excluded by stage

Two authors independently performed the selection process for each paper identified through database search or snowballing procedures. Consistent with PRISMA 2020 checklist, [20] eligibility was appraised by screening papers at three different stages: title, abstract and full text. **(**Fig. 2**)** Studies of potential interest were entered into EndNote 20 and screened at all three stages of selection.

Disagreements in the inclusion of the paper were resolved by consensus, consulting other members of the team if appropriate.

### Data extraction appraisal and synthesis

Data extraction forms were created as Excel tables. Four different tables were created corresponding to four domains of data of interest: a. general data about the paper (i.e. authors, journal, year of publication, country, type of intervention, health outcome under study, population of interest, etc.), b. intervention characteristics (i.e. description of the intervention, duration, eligibility, size of benefit, etc.) and evaluation method (including intervention and control group definition, randomisation, etc.); c. Impact and operational evidence (i.e. quantitative and qualitative findings reported); and d. Limitations of the study, including missing values, loss to follow up, and biases (as reported by the authors of the paper). In order to maximise comparability across papers, whenever possible, data were extracted in the form of multiple choice answers, true/false, and short answers. After forms were piloted, data extraction was completed by one author and subsequently validated by a second one. Given studies heterogeneity (mainly in terms of measures of impact), we did not conduct a meta-analysis. We summarised instead the principal findings of each study and combined them together via a narrative synthesis.

### Risk of bias assessment

Despite the existence of several tools for the critical appraisal of the quality of studies, we chose to use the approach suggested by Waddington et al. [18] based on the simple identification of a number of selected biases (whether explicitly stated in the papers or identified by the authors of this report). Due to the complexity and heterogeneity of the studies and their statistical techniques, in particular for quasi-experimental ones, in fact, we did not apply any bias score-based approach to determine the overall risk of bias of the eligible papers, [18] but the identified biases were listed, described, and summarised in a descriptive table (Table 6).

### Review protocol registration

The review protocol has been registered within PROSPERO in June 2020 with the registration number CRD42020178543 [21].

## Results

The search strategy returned a total of 11,658 papers. After removing duplicates and titles of no relevance, we obtained 358 papers to submit for abstract screening of which 95 were considered suitable for the eligibility assessment. Furthermore, the snowballing approach returned us 71 papers, of which 30 were considered of potential interest and read entirely to assess eligibility. Overall l 150 papers underwent eligibility assessment of which 16 met the review requirements. 134 were excluded because they did not meet one or more of the following inclusion criteria:: the quasi experimental or RCT study design; the income support intervention; the specific physical and mental health outcome of interests; the appropriate age of the study population and the high income countries context **(**Fig. 2)**.**

### Studies description

Table 2 provides an overview of the main features of the studies included in this review. Their publication year ranged from 2001 to 2019 and the considered interventions were delivered between 1957 and 2013.

**Table 2:** Synopsis of studies included in the review

*Author*	*Year of intervention implementation*	*Country*	*Type of intervention*	*Health Outcome*	*Study design*
Almond et al [34]	From 1957 to 1977- from the implementation year in the first state to all US states completed	United States	Conditional cash transfer	Birth weight^1^	Natural experiment- fixed effect model
Baker et al [38]	The reform under study occurred in 1993	United states	Earned Income Tax Credit	Birth weight^1^	Natural experiment- difference in difference and triple differences
Brownell et al [23]	Benefit was introduced in 2001	Canada	Unconditional Cash Transfer	Birth weight^1,3,4^	Natural experiment—Propensity score matching
Chung et al [33]	1982 and 1983	Alaska	Universal Unconditional Cash transfer	Birth weight^1^	Natural experiment- difference in difference
Leyland et al [22]	From April 2009 to January 2011	Scotland	Universal Unconditional Cash transfer	Birth weight^1^	Natural experiment – Interrupted time-series analysis
Hamad et al 2015 [29]	From 1986 to 2000	United States	Earned Income Tax Credit	Birth weight^1^	Panel data with Instrumental Variable strategy
Hamad et al 2016 [30]	From 1986 to 2000	United States	Earned Income Tax Credit	Child mental health^2^	Panel data with Instrumental Variable strategy
Hoynes et al [31]	1993	United States	Earned Income Tax Credit	Birth weight^1^ Small-for-gestational age^3^	Natural experiment- before and after analysis
Komro et al 2019 [26]	From 1994 to 2013	United States	Earned Income Tax Credit	Birth weight^1^	Natural experiment- difference in difference
Komro et al 2016 [36]	From 1980 to 2011	United States	Minimum Wage Salary	Birth weight^1^	Natural experiment- difference in difference
Morris et al [24]	From 1992 to 1995	Canada	Conditional cash transfer	Child mental health^2^	RCT
Milligan et al [25]	2001	Canada	Unconditional cash transfer	Social and motor development Child mental health^2^	Natural experiment- difference in difference
Rosenthal et al [35]	From 1998 to 2001	United States	Conditional cash transfer	Birth weight^1^	Panel data analysis study with Instrumental Variable analysis
Strully et al [27]	From 1980 to 2002	United States	Earned Income Tax Credit	Birth weight^1^	Natural experiment- difference in difference
Wehby et aL [37]	From 1989 to 2012	United States	Minimum Wage Salary	Birth weight^1^	Natural experiment- difference in difference
Wicks Lim et al [32]	From 1997 to 2010	United States	Earned Income Tax Credit	Birth weight^1^	Natural experiment- difference in difference

1. Measured as birth weight in g or as presence/rate of low birth weight (< 2500 g)

2. This refers to child mental health as parent -or-child reported or mental health standard measures that varied across studies: Behaviour Problems Index (BPI) in; [30] BPI-like and PBS-like scales used for National Longitudinal Survey of Children and Youth Canada (NLSCY) in; [24] Scales assessing Anxiety and separation anxiety and physical and indirect aggression in [25]

3. Small for Gestational Age- SGA- (< 10th percentile of birth weight for gestational age. Large for Gestational Age –LGA- (> 90.th percentile) [31]

With the exception of Leyland et al. [22] that provided evidence from the United Kingdom, all the other studies were conducted in North America: largely from the United States and in four cases from Canada [23–27].

The interventions largely focused on a United States poverty alleviation strategy, the EICT (Earned Income Tax Credit (seven papers) [26–32] followed by unconditional cash transfer interventions (five papers), [22, 23, 25, 33], conditional cash transfers (three papers), [24, 34, 35] and minimum wage salary (two papers) [36, 37].

Table 3 provides a detailed description of the interventions included in this review both in terms of benefits provided and recipients (i.e. the ideally target population).

**Table 3:** Intervention description by type, theory of change and target population

A*uthor*	*Intervention*	*Description of Intervention*	*Population Targeted*
Almond et al [34]	Conditional cash transfer	Food Stamp Program is the most expansive of US Food and Nutrition Program. Benefits are an important part of the program and they vary in amount through time (ex. around 200$ per recipient household per month in 2011) and can be used to purchase all food items The absence/presence of this program at county level was used to study its effect on birth weight	Eligible low income US residents
Baker et al [38]	Earned Income Tax Credit	The Earned Income Tax Credit (EITC) is the largest poverty alleviation program in the US, It involves a tax rebate to low-income families contingent upon their employment, with larger benefits for recipients with children. Individuals with no earned income are not eligible. The size of the credit increases with increasing earned income, eventually plateauing followed by a phase-out of benefits. Initiated in 1975, the program was expanded in 1993, creating substantial variation in the size of the tax credit awarded to recipients. Individual states also offered differing amounts of earned income tax credits that underwent expansions during the study period. The quasi-random nature of these variations – in that they are unassociated with individual characteristics – presents the opportunity to more clearly identify the impacts of the EITC on health	Low-income families in the United States contingent upon having an earned income
Brownell et al [23]	Unconditional Cash Transfer	Healthy Baby Prenatal Benefit is an unconditional cash transfer of max 81.41 Can$ per month given during the second and third trimesters of pregnancy. It was given monthly upon request if they fit eligibility criteria (documented annual incomes below Can$32,000 and pregnancy confirmed by a physician)	All women receiving welfare during pregnancy in Manitoba province- Canada
Chung et al [33]	Universal Unconditional Cash transfer	The benefit is a fund dividend, provided from Alaska’s government, to each Alaska’s resident. It is an unconditional cash transfer that was given for two years: 1982 and 1983. The amount was of $1,000 per person in nominal dollars in 1982 $386.15 per person in 1983	All residents in Alaska in 1982 and 1983
Leyland et al [22]	Conditional cash Transfer	The HiP grant was a universal conditional cash transfer of £190 for women reaching 25 weeks of pregnancy if they had sought health advice from a doctor or midwife. It was intended to provide additional financial support in the last months of pregnancy to contribute towards a healthy lifestyle. The grant was introduced for women with a due date on or after 6 April 2009 and subsequently withdrawn for women reaching the 25th week of pregnancy on or after 1 January 2011	All women in Scotland (but the intervention was delivered through all Great Britain and Northern Ireland from 2009 to 2011) reaching 25 weeks of pregnancy if they had sought health advice from a doctor or midwife
**Hamad et al 2015** [29]	Earned Income Tax Credit	Same as described by Baker et al	Low-income families in the United States contingent upon having an earned income
Hamad et al 2016 [30]	Earned Income Tax Credit	Same as described by Baker et al	See above
Hoynes et al [31]	Earned Income Tax Credit	Same as described by Baker et al	Single mothers aged 18 and older with singleton births
Komro et al 2019 [26]	Earned Income Tax Credit	Same as described by Baker et al	Single mothers aged 18 and older with singleton births
Komro et al 2016 [36]	Minimum Wage Salary	The minimum wage for workers is the lowest remuneration that employers can legally pay their employees. In the United States it is regulated both at state-level and federal level. The laws underwent several changes in time and this study examined relative changes in law and amount by month from 1980 through 2011, calculating the difference between state-level minimum wage and the federal minimum wage in each state and month. All calculations have been adjusted for inflation by expressing all differences in 2011 dollars	All United States population- in particular it affects low educated individuals
Milligan et al [25]	Unconditional cash transfer	Starting in 1998, the core Canada Child Tax Benefit was augmented with a new program called the National Child Benefit. Under the National Child Benefit program, the federal government provided a cash benefit called the National Child Benefit Supplement (NCBS). By 2008, the NCBS reached monthly rates of Canadian $169 for a first child, $149 for a second, and $142 for subsequent children In 2001, the province of Manitoba changed its approach to the NCBS. Prior to 2001, Manitoba was one of the provinces that reduced welfare checks when a family received the NCBS, dollar for dollar. However, starting in 2001, Manitoba ended this “clawback” for children age zero to five. Furthermore, in 2003, the clawback exemption was extended to all children age zero to eleven. This policy reform implied an increase in income for families. Also the receipt of the NCBS check was not conditional upon parents employment	Low-income families eligible for the NCBS checks. Authors focussed on all children aged 0 to 5 years between the years 1999 and 2005. Years from 2001 onward were coded as being “after” the policy change
Morris et al [24]	Conditional cash transfer	The Self-Sufficiency Project (SSP)was a demonstration program designed to make work a viable alternative to welfare for low-income parents, whose skills and experience would likely relegate them to low-paying jobs. SSP’s financial supplement paid parents who left welfare and worked at least 30 h per week half the difference between their actual earnings and a target level of earnings. The target earnings were set at Can$30,000 in New Brunswick and Can$37,000 in British Columbia a year	Single parents in British Columbia and New Brunswick who had been on welfare for at least a year were selected at random from the welfare rolls between November 1992 and March 1995
Rosenthal et al [35]	Conditional Cash Transfer	In November 1999, Las Vegas introduced a program to encourage members to seek prenatal care in the first trimester of pregnancy to complement its traditional high-risk maternity management program. The program offered US$100 to both the pregnant member and the member’s network obstetrician or midwife after delivery upon verification that the patient entered care during the first trimester and completed regular visits thereafter	Pregnant women enrolled in the program from 1998 to 2001
Strully et al [27]	Earned Income Tax Credit	Same as described by Baker et al	Low-income families in the United States contingent upon having an earned income
Wehby et al [37]	Minimum Wage Salary	The minimum wage for workers is the lowest remuneration that employers can legally pay their employees. In the United States it is regulated both at state-level and federal level. The laws underwent several changes in time and this study examined changes in amount by month from 1989 to 2012 Over the sample period (1988–2012), the federal minimum wage increased from $3.35 to $7.25 and they examined the real ($2012) minimum wage, which is the nominal wage deflated by the consumer price index	All United States population- in particular it affects low educated individuals
Wicks-Lim et al [32]	Earned Income Tax Credit	Same as described by Baker et al. but the study focus only on New York state expansion and examined the effects at neighbourhood level	Low income neighbourhood in Yew York City

In terms of child health outcomes (Table 2), the vast majority of studies focused on birth weight as both its measurement in grams and Low Birth Weight (LBW) percentage. In two studies, [23, 31] also weight-for-gestational age was examined. In three cases, authors focused on child mental health through specific instruments, namely the Behavior Problem Index (BPI), [30] physical/indirect aggression score and separation anxiety score [25].

Studies have largely relied on quasi-experimental study designs, whereas RCTs have been considered only in one conditional cash transfer from Canada **(**Table 3**)** [24]. Quasi-experimental designs adopted a wide range of impact evaluation techniques of different rigour and complexity: before and after analysis, [31] difference in difference, [25–28, 32, 33, 36, 37] instrumental variable analysis, [29, 30] interrupted time series analysis, [22] propensity score matching, [23] and Fixed Effect Model [34].

### Effect findings

We reported impact findings both qualitatively (Tables 4 and 5) and quantitatively (Table S2): if the considered paper provided one main model we referred to it, while if the study adopted multiple models or subgroups, all findings’ directions were reported in the tables.

**Table 4:** Effect findings by study design and type of intervention

*Intervention*	*Study design*	*Positive impact*^*1*^	*Negative impact*^*2*^	*Null effect*
***Unconditional cash transfer***				
Brownell et al. [23]	Natural experiment—Propensity score matching	•	•^a^	
Chung et al. [33]	Natural experiment- difference in difference	•		
Leyland et al. [22]	Natural experiment – Interrupted Time Series analysis			•
Milligan et al. [25]	Natural experiment – Difference in Difference	•		
***Conditional cash transfer***				
Morris et al. [24]	RCT*			•
Rosenthal et al. [35]	Natural experiment – IV* analysis	•		
Almond et al. [34]	Natural experiment- fixed effect model	•		
***Earned income tax credit***				
Baker et al. [38]	Propensity score matching	•		
Hamad et al. 2015 [29]	Natural experiment—IV analysis			•
Hamad t al. 2016 [30]	Natural experiment—IV analysis	•		
Komro et al. 2019 [26]	Natural experiment – Difference in Difference	•		
Hoynes et al. [31]	Natural experiment – Before and After	•		
Strully et al. [27]	Natural experiment- difference in difference	•		
Wicks-Lim et al. [32]	Natural experiment- difference in difference	•		
***Minimum wage salary***				
Komro et al. 2016 [36]	Natural experiment- difference in difference	•		
Wehby et al. [37]	Natural experiment- difference in difference	•		

1. Effect in the expected direction (i.e. health outcome improved after the intervention).

2. Effect in the opposite direction expected (i.e. health outcome worsened after the intervention)

a. For Large for Gestational age only an increased risk was found

^*^RCTs, randomised controlled trials; IV, instrumental variable

**Table 5:** Effect findings by child health outcome

*Child outcome*	*Intervention*	*Positive impact*^*1*^	*Negative impact*^*2*^	*Null effect*
***Birth weight***				
Almond et al. [34]	Conditional cash transfer	•		
Baker et al. [38]	Earned Income Tax Credit	•		
Brownell et al. [23]	Unconditional cash transfer	•	•^a^	
Chung et al. [33]	Unconditional cash transfer	•		
Leyland et al. [22]	Unconditional cash transfer			•
Hamad et al. 2015 [29]	Earned Income Tax Credit			•
Hoynes et al. [31]	Earned Income Tax Credit	•		
Komro 2019 et al. [26]	Earned income Tax Credit	•		
Komro 2016 et al. [36]	Minimum wage salary	•		
Rosenthal et al. [35]	Conditional Cash Transfer	•		
Strully et al. [27]	Earned income Tax Credit	•		
Wehby et al. [37]	Minimum Wage Salary	•		
Wicks-Lim et al. [32]	Earned income Tax Credit	•		
***Child mental health***				
Hamad et al. 2016 [30]	Earned Income Tax Credit	•		
Morris et al. [24]	Conditional cash Transfer			•
Milligan et al. [25]	Unconditional cash transfer	•		

1.Effect in the expected direction (i.e. health outcome improved after the intervention)

2.Effect in the opposite direction expected (i.e. health outcome worsened after the intervention)

a.In Large for Gestational age only an increased risk was found

Findings have been labelled as positive, when demonstrating a positive effect in the expected direction (i.e. health outcome improvement), negative when showing an impact in opposite direction expected (i.e. health outcome worsening) or null, when no effect or any clear direction was observed. In the labelling process we did not consider the issue of statistical significance alone, but we evaluated p values and Confidence Intervals (CI) (where available) of the estimates and integrated them in the study context and with the overall conclusions of the authors. This was further due to the fact that some of the studies performed analyses across different subgroups, therefore more than one model was presented and a more integrated interpretation was needed throughout the labelling process.

As shown in Table 4 and 5, for almost all the considered interventions results seem to lean toward an overall positive effect on the considered outcome, with some exceptions. Null results were observed in three studies. In one case [23] authors documented a negative impact of the intervention: in Brownell et al. an increased risk of Large for Gestational Age (LGA) of 1.13 (95% CI: 1.05–1.23) was found for children whose families received an unconditional cash transfer during pregnancy in Canada with respect of families who did not receive it. However, these results were paralleled with positive findings in other outcomes as a decreased risk of LBW (Relative Risk RR: 0.71, CI 0.63–0.81) and Small for Gestational Age (SGA) (RR: 0.90, CI 0.81–0.99) [23].

### Findings by intervention type

Table 4 provides a distribution of the impact findings by type of intervention. No effect on mental health was found for the RCT on the Self Sufficiency Project (SSP), [24] a conditional cash transfer aimed to increase employment, which provided a financial supplement to parents who left welfare and worked at least 30 h per week.

EITC, instead, a tax rebate on earned income for low income families of the United States, [26, 27, 29–31, 37, 38] seems to produce almost consistently a positive impact on child birth weight. With the exception of Hamad et al., [29] all remaining quasi-experimental studies showed a positive impact of this intervention on child-birth weight among recipients compared to those not receiving these benefits.

Finally, the remaining unconditional and conditional cash transfer interventions examined showed overall a positive impact on child health, both on reduction of absolute birth or LBW [23, 33–35] and on children’s mental health scores [25].

### Findings by study design and health outcomes

We did not have enough evidence to assess the robustness of results to the study design**(**Table 5**)**. The only RCT included in this review showed a null effect of the intervention on child mental health when comparing beneficiaries and non-beneficiaries of the intervention [24, 25]All quasi-experimental studies, except for Milligan et al. [25] and Hamad et al., [29] found at least one positive effect.

Despite the differences in the number of studies tackling birth weight and mental health (i.e. respectively 13 vs 3), evidence seems to suggest a more consistent response from studies looking at birth weight compared to those looking at mental health (Table 5).

Specifically, studies looking at mental health showed a more mixed picture, with positive and null effects almost equally represented among studies: the RCT, concerning the SSP program, designed to promote work among low-income families did not find any effect on mental health. Conversely the other two quasi-experimental studies concerning respectively the EITC [30] and the Canadian Child Tax Benefit, [24] found a positive effect of the interventions on children's mental health when comparing beneficiaries with non-beneficiaries. Evidence on birth weight offered better consistency with findings being more aligned and showing overall a clearer and more consistent positive impact among beneficiaries in most studies included in this review.

### Magnitude of positive effects

All quantitative findings from the studies included in this review are available in the online appendix Additional file 2; however, a comparison across them was not always feasible. For example, when looking at the quantitative findings for mental health outcomes, the different mental health outcomes considered and the different measurement approaches taken hampered any meaningful comparison.

As for birth weight, even if for this outcome the direction of results was more consistent, the magnitude of impact varied among studies and according to the type of intervention implemented. However, most of the EITC studies [26, 31, 32, 38] found a decline of LBW rate in the general population ranging from 1.6% [31] to more than 10% [26] among children benefitting from the intervention. The study from Komro et al. 2019 [26], which stratified results by participants’ ethnicity, found also that the increase of absolute birth weight in EITC recipients' newborns was proportional to the extent of the perceived benefit, he increase in birth weight ranged from 8.6 g for non-Hispanic women receiving a non-refundable tax credit lower than the 10% of the federal amount), to 37.1 g for newborns of Black women receiving a refundable tax credit higher than the 10% of the federal amount.

Because of the high heterogeneity of studies involved and the modest impact size, also the population health implications remain uncertain. However, in two cases, [26, 36] authors attempted to extrapolate the observed effect into actual negative public health outcomes averted. According to Komro et al. 2019, [26] the 12% reduction in LBW produced by the EITC translated into 3,760 fewer LBW babies born from Black mothers and 8,364 fewer LBW babies born from White mothers per year across the United States. Hispanic and non-Hispanic mothers displayed relatively similar effects. For minimum wage salaries instead, if all United States in 2014 had increased their minimum wages by 1 dollar there would likely have been an estimated 2,790 fewer LBW births for the year [36].

### Conceptual frameworks

The majority of papers included in this review, except for three, [23, 32, 36] explicitly mentioned a theory of change or logic model either informing their study hypotheses or guiding the results interpretation. Multiple pathways were speculated through which those interventions, aimed at income support, could affect perinatal health if delivered during pregnancy.

Health-related behaviours were predominantly mentioned, mainly smoking, alcohol, and consumption of unhealthy foods that are unevenly distributed across different socioeconomic positions. Those behaviours can directly affect infant health, acting in particular on intra-uterine growth that eventually is a key determinant of birth weight [34].

In addition, women with lower household income suffer from higher rates of malnutrition, demonstrate heightened psychological stress associated with neuroendocrine dysfunction, which can ultimately influence the likelihood and duration of breastfeeding and hamper access to adequate prenatal care services [29] In particular, maternal healthcare utilisation behaviours (prenatal care) was analysed in three studies [31, 33, 35] suggesting some evidence for a mediating role.

Some studies also mentioned the “family process” conceptual model, [24, 26, 27, 29–31] which postulates that the extra income provided by child benefits may improve long-run outcomes, not only through direct investments but by improving also the emotional environment in which the children grow. Specifically, maternal depression and parental warmth were both identified as potential mediators of welfare programs’ impact in most studies [24, 26, 27, 29, 37, 39] In all of these, income and employment were hypothesised to affect parental mental health which in turn affected child physical and mental health.

### Quality assessment of the studies

In all the assessed studies, at least one bias type was detected. According to Waddington et al., [18] we reported on Table 6 the identified biases if declared in the paper or detected by one of the authors of this review. Studies often dealt with complex analyses and multiple statistical tools. However, due to the complexity of evaluating such interventions, the majority of the studies were at risk of exposure misclassification, either differential or non-differential. Finally, most of the studies were affected by incomplete reporting either because of the incomplete sharing of study results or, in most cases, the lack of information critical for the interpretation of study findings (e.g. how they dealt with missing data).

**Table 6:** Biases identified in the studies included in the review

	**Information bias**	**Misclassification**	**Omitted variable bias**	**Reporting bias**	**Selection bias**	**Other**
***Description***	Differences in the collection, recall, recording or handling of information used in a study	Incorrect classification of participants into categories	The statistical model leaves out one or more relevant variables	Selective disclosure or withholding of information by parties involved in the design, conduct, analysis, or dissemination	Individuals or groups in a study differ systematically from the population of interest	
Almond et al. [34]		•		•		
Baker et al. [38]		•	•			
Brownell et al. [23]		•			•^a^	•^a^
Chung et al. [33]	•	•				
Hamad et al. 2015 [29]	•	•			•	•^b^
Hamad et al. 2016 [30]	•	•				
Hoynes et al. [31]		•	•	•		
Komro et al. 2016 [36]		•		•		
Komro et al. 2019 [26]					•	•^c^
Leyland et al. [22]	•			•		
Milligan et al. [25]				•	•	
Morris et al. [24]				•		
Rosenthal et al. [35]			•	•		
Strully et al. [27]		•				•^d^
Wehby et al. [37]		•		•		
Wicks-Lim et al. [32]	•	•				•^e^

a. They could not ascertain how much unmeasured confounding influenced results, causing endogeneity bias. Furthermore, they limited the evaluation to women receiving welfare rather than examining all low-income women receiving the income supplement during pregnancy, thus limiting the generalizability of results

b. Recall bias may be present from women interviewed not in the year of childbirth

c. Observed result of LBW could be an underestimation of the differential effect by race because black women had a double rate of stillbirths

d. Mother's life environment and attitude or unmeasured genetic variation could influence results

e. Some factors that could cause spurious correlation such as neighbourhood gentrification, were impossible to control for

## Discussion

This review aimed to quantify the impact of income-support strategies on life-course risk factors and health outcomes. This review adds to the existing literature by providing insights on the impact of specific types of macro-economic interventions on a specific window period (i.e. first 1,000 days of life) on a selected list of specific life-course risk factors and health outcomes. Consistent with similar evidence synthesis efforts, [40] in this review we could not conclusively demonstrate an effect of income-support-strategies on all the selected child outcomes. Overall, evidence available suggests that income support strategies have a positive, albeit small, effect on birth weight and limited impact on mental health indicators. No other health outcome of interest was investigated in the studies included in this review, so no inference can be made on cardiometabolic and respiratory health outcomes.

One could argue that despite the small observed effect, the proportion of people exposed to these policies is quite large which could result overall into a considerable effect in public health terms. Nonetheless, only two studies in this review have tried to extrapolate this effect to the population level [27, 31].

The general conclusion of this review seems to be robust to the type of intervention and child outcome under observation.

There are possible, not mutually exclusive, explanations for the results of our review, including the fact that despite the extensive screening of different, multidisciplinary literature browsers, the search strategy returned a relatively small number of eligible studies. This is consistent with the conclusions of similar previous reviews [40] which already underlined the scarcity of evidence. In fact, very few experimental or quasi-experimental studies have been undertaken to explore the impact of complex, macro-level socioeconomic interventions on child health and—even less—on specific, well measured child health outcomes. We restricted the review to well-defined life-course risk factors and health outcomes, thus excluding studies assessing the effects on generally self-reported health or well-being. Those subjective outcomes are in fact more likely to be affected by both non-differential and differential misclassification. This selection has, however, further limited the literature to draw upon. The limited effect on mental health outcomes could be also due to the poor standardisation in the definition and measurement of these outcomes.

Some authors [41, 42] argued that quasi-experimental studies may be more suitable to the evaluation of complex interventions with weak effects in a large group of the population. Given the limited number of RCTs included in our review, this remains speculative, but there is clearly the need to understand how methodological aspects influence our understanding and measurement of the health impact of these policies. It is difficult though to establish upfront the superiority of one study design over another in the evaluation of these programs. Many factors are likely to influence the appropriateness of different methodological approaches and one could conclude that the complexity of these interventions can be only captured through a combination of qualitative and quantitative studies.

It is worth noting that with few exceptions, [22, 35] most of the interventions included in the review were not originally designed and implemented to evaluate nor achieve a health effect. This implies that some of the potential impact of these programs could have been missed purely for design/implementation reasons. On the other hand, for those interventions that had a quantifiable effect (e.g. the EITC), one could argue how bigger this effect could have been if these programs were designed with the precise intent of improving people health other than just socioeconomic measures.

The relative modest effect observed in the studies included in this review could be attributed to the size of the benefits provided: this may explain for example why the impact of the EITC (where the size of cash received can be relatively high [30, 39]) seems almost consistently positive. By contrast, the two studies on conditional cash transfers included in this review, involving a fairly small overall cash transfer provided to beneficiary women of 190GBP or 100 USD, found no evidence of an effect [22, 35]. These observations are consistent with what reported in other reviews similar to this one. For example, Lucas et al. [40] concluded that the monetary value of many interventions was low, as in most studies included in their review the total increase in income to intervention families was less than US$50 per month despite the fact that many parents were compelled to work full-time. Authors questioned whether the level of income increase was sufficient to affect living conditions and – we would add – it was big enough to ensure this effect translated into a health effect [40].

Most of the interventions included in this review focus on indicators of socioeconomic position or—broadly speaking – econometric concepts of disadvantage. While the association between these constructs and child health is widely acknowledged, this relationship is likely to be complex and mediated by a number of underlying known and unknown pathways. Importantly, if the effect on income does not translate into a tangible effect on these mediators then the expected impact on child health may not materialise as expected. Two of the included studies [25, 31] suggest a ‘family process’ mediation pathway, according to which the extra income provided by the child benefits may improve in the long-run outcomes not only through direct financial investment, but also by improving the emotional environment in which children grow up. Another important mediator is whether the increase of income happens via the mother’s employment [31]. Some authors speculated that some policies that incentivise maternal employment may involuntarily increase maternal stress and add extra pressure on mothers, which offsets the benefit of a better income on children. Similarly, Morris et al. [24] argue that a proper evaluation of the impact of better income and parental employment on child health should account for the moderating role of the developmental period of the child. According to these authors, [24] the effect of income and employment on children aged 1 or less may be counterbalanced, if not reverted, via prolonged periods of time of maternal absence that ultimately leads to increased instability of care and reduced parental warmth [24].

Our review presents with a number of limitations. Despite our comprehensive search of the literature, the evidence we gathered provides at most a partial representation of existing macroeconomic policies. This is mostly due to the limited number of health outcomes under investigation and to the heavy predominance of studies from North America, largely focussing on EITC in the United States. This unbalance is probably largely due to the fact that EITC is the most important poverty-alleviation strategy in the United States and it is particularly suitable for quasi-experimental impact evaluations because of variation in the distribution of benefits and changes in welfare policy. While our findings are still relevant, their external validity to countries beyond the United States, to different types of interventions, and other health outcomes remain limited.

Not only we found evidence just on birth weight and mental health outcomes, but those outcomes were consistently evaluated though quasi-experimental designs, with a scarce representation of RCTs.

Another limitation of the review is that we cannot exclude the extent to which the observed findings may have been distorted by historical trends of the health outcomes of interest.

In the United States, for example, LBW rates changed over the past years: after a stable period between 1950 and 1960, they increased to around 7% in the early 1990s and 7.6% in 2000 [43]. Similarly, increasing trends were observed for mental disorders, especially in high income countries such as North America [43]. These changes, when not adequately controlled for, may to some extent have counterbalanced the positive effect of the intervention seen in some of the included studies [43].

### Implications for future research

This review provides a useful contribution to the literature on the health effects of social policies. Through the extensive review of the evidence, this research allowed to speculate about possible mechanisms through which these policies may play an effect and why they seem to fail in other circumstances. Finally, through the identification of persisting knowledge gaps, it allowed to draw a research agenda for the future:

First, there is clearly a scope to invest more in the evaluation of the child health impact of macro-level socio economic interventions by financing more impact evaluations and by advocating for a better design and implementation of these policies to allow their proper health impact assessment.

Second, the association between income and child health is amply demonstrated. If interventions aiming at improving income do not obtain a commensurate effect on child health outcomes, there is clearly something not working either in the type of intervention provided or in the way we measure this effect. Quasi-experimental studies are often imperfect tools that only allow for comparisons between sub-optimal groups. On the other hand, RCTs are considered to be often unfeasible, and unethical and unable to capture the complexity of social ‘experiments’. [44] Given the above, there is a mandate to investigate the role of alternative methodologies including observational studies as well as mathematical modelling (i.e. microsimulations) in filling the numerous knowledge gaps still surrounding the impact of socioeconomic interventions on child health.

Thirdly, the question of ‘what works?’ should be more correctly replaced by ‘what works for whom and why?’. There is an urgent need to unpack the effect of these interventions to understand better the reasons for their failure and success. This could be achieved through the design of impact evaluations adopting mixed-methods approaches and/or requiring the collection of data to perform rigorous moderation and mediation analyses. This could elucidate why some sub-groups may most benefit from the intervention and through what underlying pathway. Alternatively, and perhaps more conveniently, one could complement reviews like this one, with a “realist” approach. This is a type of literature review in which evidence are mapped against a pre-defined conceptual framework, in order to validate or reject the existence of the speculated underlying pathways linking the interventions with the outcomes of interest [42]. This lens could be applied to the subject of this review and provide important additional explanations on the likely impact of these interventions on child health.

Finally, there is scope to expand this literature review by adding evidence on the long-term impact of these interventions. To the best of our knowledge, only few studies have explored the long-term health impact of income support strategies. Studies available [39, 45, 46] show consistently a long-term positive impact of the interventions of interest on all health and financial outcomes investigated. Nonetheless, due to the paucity of data, conclusions have to be drawn cautiously. It is also worth exploring the extent to which the way vaster literature from low and middle income can contribute to the understanding of the potential public health impact of income support strategies in high income countries. In other words, there may be merits in creating more connections between low/middle income and high income countries on socioeconomic interventions and explore how lessons can be extrapolated to both environments [47].

## Conclusions

On the basis of this review, we have not been able to establish conclusively whether income support policies delivered in the first 1,000 days of life are able to improve important life-course risk factors and child health outcomes. If we concentrate on birth weight, investigated through quasi-experimental studies only, evidence suggests a modest positive effect of these policies. However, the breadth and scope of the literature needs to be enriched with additional diversified evidence (in terms of health outcome, country, intervention, and other relevant contextual factors) before a definitive conclusion can be reached and the public health potential of these policies is fully understood. The association between lower income and poorer outcomes across all dimensions of child health is strong and consistent across countries and time. The fact that a relatively small number of interventions show a small or null effect should be considered as a “research call” to undertake more and better impact evaluations of these policies. There is the urgent need not only to quantify their effect, but also to provide evidence on what works best, for whom, at what development stage and—most importantly – why.

The authors further created a glossary which provides a clear operational definition for the most technical terms used throughout our review, reported in Appendix 3.

### Supplementary Information

**Additional file 1: Table S1.** Final search strategy adopted in this review.

**Additional file 2: Table S2.** Magnitude of positive effect and results interpretation^a^.

**Additional file 3: Appendix 3.** Glossary: operational definitions.

## Data Availability

Data sharing is not applicable to this article as no databases were generated or analysed during the current study.

## Abbreviations

RCT: Randomized Controlled Trial
EITC: Earned Income Tax credit
LBW: Low Birth Weight
CI: Confidence Interval
