# Supplementary material for: The impact of income-support interventions on life course risk factors and health outcomes during childhood: a systematic review in high income countries
Source: BMC Public Health. 2023 Apr 22;23:744. doi: 10.1186/s12889-023-15595-x (PMC10121417; doi:10.1186/s12889-023-15595-x)
Supplement: Supplementary file 1 — Additional file 1: Table S1. Final search strategy adopted in this review. [file 12889_2023_15595_MOESM1_ESM.pdf]

Table S1 – Final search strategy adopted in this review

Search*		Medline	Embase	Psychinfo
#1	Interventions	social programmes OR social protection OR social assistance OR government programmes OR government transfers OR cash transfers OR cash OR food OR in-kind transfers OR benefits	social programmes OR social protection OR social assistance OR government programmes OR government transfers OR cash transfers OR cash OR food OR in-kind transfers OR benefits	social programmes OR social protection OR social assistance OR government programmes OR government transfers OR cash transfers OR cash OR food OR in-kind transfers OR benefits or Psychosocial
#2		child grants OR child benefits OR child allowances	child grants OR child benefits OR child allowances	child grants OR child benefits OR child allowances
#3		Tax benefits OR Tax exemptions OR Tax credit OR Fiscal	Tax benefits OR Tax exemptions OR Tax credit OR Fiscal	Tax benefits OR Tax exemptions OR Tax credit OR Fiscal OR credit
#4		Income support OR Minimum OR Wage	Income support OR Minimum OR Wage	Income support OR Minimum OR Wage
#5		Welfare OR Social Policies	Welfare OR Social Policies	Welfare OR Welfare State OR Social Policies
#6		OR #1 to #5	OR #1 to #5	OR #1 to #5
#7	Health outcome	Health OR Resp* Health OR Wheezing OR Resp* Infections OR Mental OR Behav* OR Psycho* OR Cardio* OR BMI OR Weight OR Obesity OR Blood Pressure OR Lipid* OR Glycem*	Health OR Resp* Health OR Wheezing OR Resp* Infections OR Mental OR Behav* OR Psycho* OR Cardio* OR BMI OR Weight OR Obesity OR Blood Pressure OR Lipid* OR Glycem*	Health OR Resp* Health OR Wheezing OR Resp* Infections OR Mental OR Behav* OR Psycho* OR Cardio* OR BMI OR Weight OR Obesity OR Blood Pressure OR Lipid* OR Glycem*
#8	Population	Prenatal OR Antenatal OR Perinatal OR pregnancy OR pregnant OR mother* OR parent*	Prenatal OR Antenatal OR Perinatal OR pregnancy OR pregnant OR mother* OR parent*	Prenatal OR Antenatal OR Perinatal OR pregnancy OR pregnant OR mother* OR parent*
#9		Postnatal OR Perinatal OR Postpartum	Postnatal OR Perinatal OR Postpartum	Postnatal OR Perinatal OR Postpartum
#10		“Infant" OR "Newborn" OR "Child, preschool" OR "childhood" OR "children" OR “child*”	“Infant" OR "Newborn" OR "Child, preschool" OR "childhood" OR "children" OR “child*”	“Infant" OR "Newborn" OR "Child, preschool" OR "childhood" OR "children" OR “child*”
#11		OR #8 to #10	OR #8 to #10	OR #8 to #10
#12		#6 AND #7 AND #11	#6 AND #7 AND #11	#6 AND #7 AND #11

Search*		Scopus/Google scholar	Social Policy and Practice	Cochrane Library CENTRAL
**#1**	**Interventions**	Social protection OR social assistance OR cash transfers OR conditional cash transfers	social programmes OR social protection OR social assistance OR government programmes OR government transfers OR cash transfers OR cash OR food OR in-kind transfers OR benefits	social programmes OR social protection OR social assistance OR government programmes OR government transfers OR cash transfers OR cash OR food OR in-kind transfers OR benefits
**#2**		child grants	child grants OR child benefits OR child allowances	child grants OR child benefits OR child allowances
**#3**		Tax benefits OR Tax exemptions OR Tax credit OR Fiscal	Tax benefits OR Tax exemptions OR Tax credit OR Fiscal	Tax benefits OR Tax exemptions OR Tax credit OR Fiscal
**#4**		Income support OR Minimum Wage	Income support OR Minimum OR Wage	Income support OR Minimum OR Wage
**#5**		Welfare OR Social policies	Welfare OR Social Policies	Welfare OR Social policies
**#6**		OR #1 to #5	OR #1 to #5	OR #1 to #5
**#7**	**Health outcome**	Health OR Resp* Health OR Mental OR Behav* OR Psycho* OR Cardio* OR BMI OR Weight OR Obesity OR Metab*	Health OR Resp* Health OR Wheezing OR Resp* Infections OR Mental OR Behav* OR Psycho* OR Cardio* OR BMI OR Weight OR Obesity OR Blood Pressure OR Lipid* OR Glycem*	Health OR Resp* Health OR Wheezing OR Resp* Infections OR Mental OR Behav* OR Psycho* OR Cardio* OR BMI OR Weight OR Obesity OR Blood Pressure OR Lipid* OR Glycem*
**#8**	**Population**	Prenatal OR Antenatal OR Perinatal OR pregnancy OR pregnant OR mother* OR parent*	Prenatal OR Antenatal OR Perinatal OR pregnancy OR pregnant OR mother* OR parent*	Prenatal OR Antenatal OR Perinatal OR pregnancy OR pregnant OR mother* OR parent*
**#9**		Postnatal OR Perinatal OR Postpartum	Postnatal OR Perinatal OR Postpartum	Postnatal OR Perinatal OR Postpartum
**#10**		Child	“Infant" OR "Newborn" OR "Child, preschool" OR "childhood" OR "children" OR “child*”	“Infant" OR "Newborn" OR "Child, preschool" OR "childhood" OR "children" OR “child*”
**#11**		OR #8 to #10	OR #8 to #10	OR #8 to #10
**#12**		#6 AND #7 AND #11	#6 AND #7 AND #11	#6 AND #7 AND #11

*With the exception of Google Scolar the search strategies above were restricted to High Income countries only according to the World Bank classification for year 2020-2021.
