# Supplementary material for: The impact of income-support interventions on life course risk factors and health outcomes during childhood: a systematic review in high income countries
Source: BMC Public Health. 2023 Apr 22;23:744. doi: 10.1186/s12889-023-15595-x (PMC10121417; doi:10.1186/s12889-023-15595-x)
Supplement: Supplementary file 2 — Additional file 2: Table S2. Magnitude of positive effect and results interpretationa. [file 12889_2023_15595_MOESM2_ESM.pdf]

Table S2 – Magnitude of positive effect and results interpretation[a]

Study	Intervention	Control	Outcome description	Measure of impact	Effect estimate	Results interpretation
Birth weight						
**Almond et al(34)**	Conditional cash transfer	Women for which FSP was *vs* was non place in the county at the beginning of third trimester.	Birth weight in grams<br><br>Low Birth Weight percentage (LBW%)	Regression coefficient<br><br>Percentage point difference	**Birth weivght**<br>(1) Whites[2]: 2.039 p<0.10 Blacks: 3-454<br>(2) Whites: 2.635 p<0.05 Blacks: 4.120<br>(3) Whites: 2.089 p<0.10 Blacks: 5.466<br>(4) Whites: 2.175 p<0.05 Blacks: 1.665<br>**LBW%**<br>(1) Whites: -0.0006 p<0.10 Blacks: -0.0015<br>(2) Whites: -0.0006 a p<0.10 Blacks: -0.0016<br>(3) Whites: -0.0006 Blacks: -0.0019<br>(4) Whites: -0.0006 Blacks: -0.0009	Results shows the intervention impact of FSP on birth weight and LBW percentage, respectively in the white and black population.<br>They found this cash transfer improved birth outcomes, effects being larger in populations at greater nutritional risk relative to Whites.
**Baker et al(38)**	Earned Income Tax Credit	Among EITC recipients the size of the EITC payment for which a family was eligible was used as a predictive variable	Birth weight in grams<br><br>LBW %	DD[1] and DDD[1] regression coefficient	**Birth weight (g)**<br>DD Model1[3] 3.63 (p>0.10)<br>DD Model2 7.30 (p<0.01)<br>DDD Model2 13.8 (p<0.01)<br>**Low birth weight %**<br>DD Model1 -0.02 (p<0.01)<br>DD Model2 -0.028 (p<0.01)<br>DDD Model2 - 0.0033 (p<0.01)	Results indicate that the 1993 expansion of EITC has been effective in increasing birth weights, though the magnitude of the effects is relatively small. The effect obtained with DDD model were more reliable- due to falsification test robustness- and they were higher in magnitude.
**Brownell et al(23)**	Unconditional cash transfer	Women who recieved welfare in pregnancy that applied and received *vs* not received the benefit	LBW rate<br>Small for Gestational Age rate (SGA, weight below 10th percentile)	Risk Ratio between who received the transfer and who did not	**LBW** 0.71 (95% CI 0.63-0.81)<br>**SGA** percentage 0.90 (95% CI 0.81-0.99)	The receipt of an unconditional income supplement by very-low income women during pregnancy was associated with reduction in LBW and Small for Gestational Age percentage, and an increase of Large for Gestational Age percentage
**Chung et al(33)**	Universal unconditional cash transfer	People living in Alaska in the benefit years *vs* people living in other US states	Birth weight in grams<br><br>LBW %	DD[1] regression coefficient	**Birth weight (g)**<br>Anticipatory[4] 17.448 p<0.05<br>Realized 30.924 p<0.05	They found that an additional $1,000 ($2,331 in 2011 dollars) increases birth weight by 17.7

		in the same period			**LBW %**<br>Anticipatory -0.004 $p<0.05$<br>Realized -0.007 $p<0.05$	g and decreased the likelihood of LBW. Though the APFD affects the overall population rather than just the poor, the effect was higher the income effect is higher for less educated mothers.

Study	Intervention	Control	Outcome description	Measure of impact	Effect estimate	Results interpretation
Birth weight						
**Hoynes et al(31)**	Earned Income Tax Credit	Model 1: second or higher vs first pregnancy<br>Model 2:second, third of higher vs first pregnancy (decomposed effect)<br>Model 3: third or higher vs first pregnancy	LBW %	DD[1] regression coefficient	Parity 2+ versus Parity 1[5]: -0.35, $p<0.01$<br>Parity 3+ versus Parity 1: -0.53, $p<0.01$<br>Parity 3+ versus Parity 2: -0.34, $p<0.01$<br>Parity 2 versus Parity 1: -0.16, $p<0.05$	For single, low-education (12 years or less) mothers, a policy-induced treatment on the treated increase of $1000 in after-tax income is associated with a 0.17 to 0.31 percentage point decrease in low birth weight status. Given roughly 10.7 percent of treated children were low birth weight, this represents a 1.6 percent to 2.9 percent decline.
**Komro 2019 et al(26)**	Earned income Tax Credit	Eligible women in state with vs without EITC in place	Birth weight in grams<br><br>LBW %<br>All results are stratified by race and shown according to the intervention characteristics[6]	DD[1] regression coefficient	<u>Birth weight</u><br>*EITC, NR, < 10%*<br>Black:16.120, $p<0.01$<br>White:9.830, $p<0.01$<br>Hispanic:11.264, $p<0.01$<br>Non-hispanic:8.667, $p<0.05$<br>*EITC, R, <10%*<br>Black: 19.342, $p<0.01$<br>White:18.219, $p<0.05$<br>Hispanic:21.431, $p<0.01$<br>Non-hispanic: 15.530, $p<0.10$<br><br>*EITC, NR, 10%+*<br>Black:19.257, $p<0.01$<br>White:10.462, $p<0.01$<br>Hispanic:16.561, $p<0.01$<br>Non-hispanic:11.851, $p<0.01$<br>*EITC, R, 10%+*<br>Black:37.164, $p<0.01$<br>White:28.400, $p<0.01$	

					Hispanic:35.613, *p<0.10*<br>Non-hispanic:28.492, *p<0.01*<br><u>LBW %</u><br>*EITC, NR, < 10%*<br>Black:-0.007, *p<0.01*<br>White:-0.002, *p<0.05*<br>Hispanic:-0.001, *p<0.10*<br>Non-hispanic:-0.004, *p<0.01*<br>*EITC, R, < 10%*<br>Black:-0.009, *p<0.01*<br>White:-0.005, *p<0.01*<br>Hispanic:- 0.004, *p<0.05*<br>Non-hispanic:-0.006, *p<0.05*<br>*EITC NR, 10%+*<br>Black:-0.006, *p<0.05*<br>White:-0.002, *p<0.01*<br>Hispanic:-0.002, *p<0.05*<br>Non-hispanic:-0.003, *p<0.05*<br>*EITC R, 10%+*<br>Black:-0.014, *p<0.01*<br>White:-0.007, *p<0.01*<br>Hispanic:-0.007, *p<0.01*<br>Non-hispanic:-0.009, *p<0.01*	
**Komro 2016 et al(36)**	Minimum wage salary	States with minimum wage higher *vs* lower than federal one	LBW percentage	DD[1] regression coefficient	**Crude[7]**: -1.9 (95% CI -3.1-0.7)<br>**Crude lagged**[8]: -2.2 (95%CI -3,6-0.8)<br>**Adjusted**: -1.1 (95% CI -2.1;-0.1)<br>**Adjusted lagged**: -1.3 (95%CI -2,7;0)	Results showed that increased state minimum wages are associated with reduced low birth weight births. The analyses were at the state level and refers to one dollar increase in minimum wage salaries
**Rosenthal et al(35)**	Conditional Cash Transfer	Women who participated in the project vs women that not participated in the program	LBW rate	Logistic regression coefficient (Odd Ratio) obtained through Instrumental Variable analysis	0.53 (95%CI: 0.23; -1.18)	Despite non-significance, the magnitude of effect is similar to the one obtained through ordinary logistic regression in which the exposure (i.e. intervention enrolment) is the one as reported in the dataset.

**Strully et al(27)**	Earned Income Tax Credit	Mothers who gave birth in their state of residence that had *vs* had not EITC in place in the given year	Birth weight in grams	DD[1] regression coefficient	15.704 ($p<0.01$)	Using a natural experimental strategy, they found that state EITCs increase birth weights in low income populations. Since they used dichotomous indicators for larger and smaller maximum state credits, they found no evidence of a dose-response effect on birth weight
**Wehby et al(37)**	Minimum Wage Salary	The amount of increase in minimum wage was used as a predictive variable	Birth weight in grams<br><br>LBW %	DD[1] regression coefficient	**Birth weight (g)**<br>1.04 $p<0.01$<br>**LBW%**<br>-0.00090 $p<0.05$	The effect of the increase of 1$ in minimum wage is the increase of 4.04 grams in birthweight and a reduction of 0.009 % in rate of LBW.<br>They estimate “affected hours” as average annual working hours so that they could estimate the effect of 1000$ in annual income. Results of estimate are an increase of 8.468 grams and a reduction in 0.002% in LBW rate, both non-significant at 95% level
**Wicks-Lim et al(32)**	Earned Income Tax Credit	Neighbourhoods which received at least $300 in EITC benefits per capita vs less impacted neighbourhoods	LBW rate	DD[1] regression coefficient	**Crude results**<br>OLS[9] model -0.021 (SE 0.008) $p<0.05$<br>GLM model -0.331 (SE 0.162) $p<0.05$<br><br>**Adjusted results**<br>OLS model -0.022 (0.008) $p<0.01$<br>GLM model -0.330 (0.173) $p<0.1$	NYS and NYC EITC expansions between 1997 and 2010 are linked to improved low birthweight rates in the city’s low-income neighbourhoods. The magnitude of the EITC’s impact on low birthweight rates suggests ecological effects, and an additional channel through which anti-poverty measures can serve as public health interventions
Child mental health						
**Hamad et al 2016(30)**	Earned Income Tax Credit	Families with 2 or more children *vs* mothers giving	BPI score[10]. The score was measured after 2 and 4 years[11]	Linear regression coefficient (BPI score difference)	**2 year difference**<br>-0.46 (95%C.I: -0.86;-0.091)<br><br>**4 year difference**<br>-0.41 (95% C.I.: -0.91;-0.001)	Results suggest that there were positive effects on children’s behavioural problems in the

		birth to their first child				sample overall. The effect magnitudes were approximately 5% of a standard deviation for every $1,000 of income. Although these associations were modest, it is possible that persistent increases in income might bring about greater cumulative changes in child development
**Milligan et al(25)**	Unconditional cash transfer	Region of implementation vs region with no impementation	Social and motor development score[12] Physical aggression score Separation anxiety score Indirect aggression score Anxiety score	Regression coefficient	Social/motor development 0.187, *p<0.01* Physical aggression -0.208, *p<0.01* Separation anxiety -0.138 Indirect aggression -0.145 Anxiety -0.204, *p<0.10*	

a. As reported by the authors in the papers.

1. Estimated through Difference in Difference (DD) and/or Triple Difference (DDD)
2. For each outcome, they report estimates from four specifications with different controls (1) includes county and time (year x quarter) fixed effects, county per capita income, REIS county-level per-capita transfers, and 1960 county characteristics interacted with linear time (2) with state specific linear time trends, (3) with unrestricted state by year fixed effects, and (4) with county specific linear time trends
3. Since EITC 1993 expansion increased both the maximum credit for all families and generated significant differences in benefits for families with 2 or more children and families with 1 child, model 1 uses mothers giving birth to their first or second child as a control (partial EITC reform effect) and model 2 uses as control mothers giving birth to their first child (full EITC reform effect)
4. The intervention group was divided in two periods, Anticipatory- born prior the intervention delivery but when they were expected to receive it, and Realized- born after individuals received the credit
5. The study used different models comparing parity of the mothers because EITC treatment corresponds to the number of children prior to the current birth. Model 1 uses women at second or higher pregnancy as intervention group and women at first as control. Model 2 uses separately women at second and third or higher pregnancy as control vs first pregnancy. Model 3 uses mother at third or higher pregnancy vs mother at second.
6. Intervention categories are defined in this way: NR/R (Non-refundable or Refundable amount) and intervention benefit size groups (reported as percentage of the federal amount, i.e. less than 10%, 10% or more). All EITC categories are compared with states with no EITC (reference).
7. Crude results are adjusted for state and year fixed effect only while adjusted results take in account also of race, poverty, cigarette sales and maternal age
8. Lagged results refers to 12 months after policy change
9. Two different models were used: Ordinary Least Square (OLS) regression and Generalised Linear Models (GLM)

10. 6 Different scales are used as Behavior Problems Index, Behavior Problems Scale, Survey Diagnostic Instrument Conduct Disorder. Result are reported as standard deviation of the scores, normalized
11. In the results BPI score difference was calculated both as 4 years after the intervention vs actual difference and 2 years after the intervention vs actual difference
12. Scales used in this study are Canadian scales of parents reports developed for use in the National Longitudinal Survey of Children and Youth. Results are stratified per age. We took in account the impact on pre-schoolers at 36 months of follow-up
