# Supplementary material for: The impact of income-support interventions on life course risk factors and health outcomes during childhood: a systematic review in high income countries
Source: BMC Public Health. 2023 Apr 22;23:744. doi: 10.1186/s12889-023-15595-x (PMC10121417; doi:10.1186/s12889-023-15595-x)
Supplement: Supplementary file 3 — Additional file 3: Appendix 3. Glossary: operational definitions. [file 12889_2023_15595_MOESM3_ESM.pdf]

GLOSSARY: operational definitions

Macro-level socio economic interventions: interventions implemented to address social and/or economic issues on a large scale thus affecting entire communities, states or countries

Life course risk factors: single or multiple conditions which might occur at any point of the life course (*i.e.* from conception onwards) able to affect lifelong health (*e.g* childhood obesity might affect cardiometabolic health in adulthood)

Income-support interventions: All measures taken by government to provide an adequate income to their citizens via different benefit schemes, which be implemented within different policies with different aims and objectives

High Income Countries: all countries with a gross national income per capita exceeding $13,205 in 2021, according to the World Bank definition

Childhood poverty: children raised in poor families (*i.e below the absolute/relative poverty line: the main poverty line used in the OECD and EU is set at 60% of the median household income*) and orphans raised with limited or no state resources

Poverty alleviation strategies: All the government's economic, structural or social policies and programs aimed at reducing poverty
